# Detection of thioredoxin-1 using ultra-sensitive ELISA with enzyme-encapsulated human serum albumin nanoparticle

**DOI:** 10.1186/s40580-019-0210-5

**Published:** 2019-12-09

**Authors:** Myeong-Jun Lee, Eun-Sol Lee, Tae-Hwan Kim, Ju-Won Jeon, YongTae Kim, Byung-Keun Oh

**Affiliations:** 1 Department of Chemical & Biomolecular Engineering, Sogang University, Mapo-Gu, Seoul, 04170 South Korea; 2 Department of Applied Chemistry, Kookmin University, Seoungbuk-Gu, Seoul, 02707 South Korea; 3 George W. Woodruff School of Mechanical Engineering, Wallace H. Coulter Department of Biomedical Engineering, Parker H. Petit Institute for Bioengineering and Bioscience, Institute for Electronics and Nanotechnology, Georgia Institute of Technology, Atlanta, GA 30332 USA

**Keywords:** Signal amplification, Nanoparticle, HRP, ELISA, Trx-1, HSA

## Abstract

Many methods for early diagnosis of the disease use biomarker tests, which measure indicators of biological state in body fluids or blood. However, a limitation of these methods is their low sensitivity to biomarkers. In this study, human serum albumin (HSA) based nanoparticles capable of encapsulating excess horseradish peroxidase (HRP) are synthesized and applied to the development of enzyme-linked immunosorbent assay (ELISA) kit with ultra-high sensitivity. The size of the nanoparticles and the amount of encapsulated enzyme are controlled by varying the synthesis conditions of pH and protein concentration, and the surface of the nanoparticles is modified with protein A (proA) to immobilize antibodies to the nanoparticles by self-assembly. Using the synthesized nanoparticles, the biomarker of breast cancer, thioredoxin-1, can be measured in the range of 10 fM to 100 pM by direct sandwich ELISA, which is 10^5^ times more sensitive than conventional methods.

## Introduction

Biomarker testing is one way to analyze infections or perform a diagnosis [1, 2]. It measures the concentration of the characteristic substances of blood or body fluids, which are produced in large quantities in a diseased person, but rarely in ordinary people. The biomarker testing does not include radiation exposure or painful procedures, so it is usually used as a diagnostic tool or as an indicator of the progress of treatment in case of suspected disease [3, 4]. Among biomarker testing methods, immunoassays are widely used because they show high selectivity and sensitivity by using antigen and antibody reactions [5]. Enzyme-linked immunosorbent assay (ELISA) is one of the main immunoassays due to its simple tool and easy colorimetric visual readout [6–13]. However, for early stage disease diagnosis and trace amounts of biomarker detection, the color change may be below the limit of detection (LOD). Thus, improving the sensitivity of ELISAs for the detection of target with very low concentrations is very important for a wide range of applications of conventional ELISAs.

Various studies have been conducted to increase the sensitivity of the ELISA. Modification of the ELISA plate surface with gold (Au) nanoparticles or polycarbonate membranes can increase the sensitivity of the ELISA because it can increase the binding ability of the proteins on the plate [14, 15]. But it takes a long time to deform the surface of the plate and the preparation is complicated. Many researchers have therefore developed nanoprobes that immobilize antibodies and large amounts of signaling enzymes on nanomaterials such as Au nanoparticles or carbon nanotubes. However, these nanoprobes have limitations in improving ELISA sensitivity because they immobilize antibodies and signaling enzymes on limited surfaces of nanoparticles. Therefore, in order to develop an ELISA kit with very high sensitivity, it is necessary to develop a novel nanoprobe that may contain an excess of signaling enzymes.

In this study, a new signal amplified nanoparticle made of human serum albumin (HSA) and horseradish peroxidase (HRP) was synthesized and used as a probe producing colorimetric outputs to detect the target proteins through chemical reaction with 3,3′,5,5′-tetramethylbenzidine (TMB) [16]. HSA commonly exists in human serum and its low reactivity makes it suitable for avoiding nonspecific reactions in human samples. Using this protein as a structure, excess HRP encapsulated HSA nanoparticles (HEH) were developed. In addition, the surface of HEH was treated with Protein A (HEHP). Protein A (proA) is used as a biological linker to couple antibodies to the surface, because it can bind the Fc region of most immunoglobulins [17]. Therefore, it is very easy to immobilize the antibodies on the enzyme-encapsulated HSA nanoparticles by self-coupling between pro A and the antibodies. The final signal-enhancing nanoparticle probe was named HEHPA.

Thioredoxin-1 (Trx1) was used as a model protein to prove the concept of signal enhanced ELISA using the developed nanoprobe. Trx1 is a relatively recently discovered breast cancer biomarker that can give you an accurate diagnosis of the initial state of breast cancer [18, 19]. Since traditional breast cancer biomarkers such as CA15-3 and CEA reflect the levels of bulky tumors, they have a limitation in use for screening and early diagnosis of breast cancer [20–22].

Here, we present a novel signal enhancement nanoprobe, HEHPA and its optimization procedure. It also shows how powerfully this nanoprobe based ELISA kit amplifies the signal for Trx1 compared to conventional ELISA kit.

## Results and discussion

### Overview of ultra-sensitive ELISA using HEHPA

**Fig. 1:**
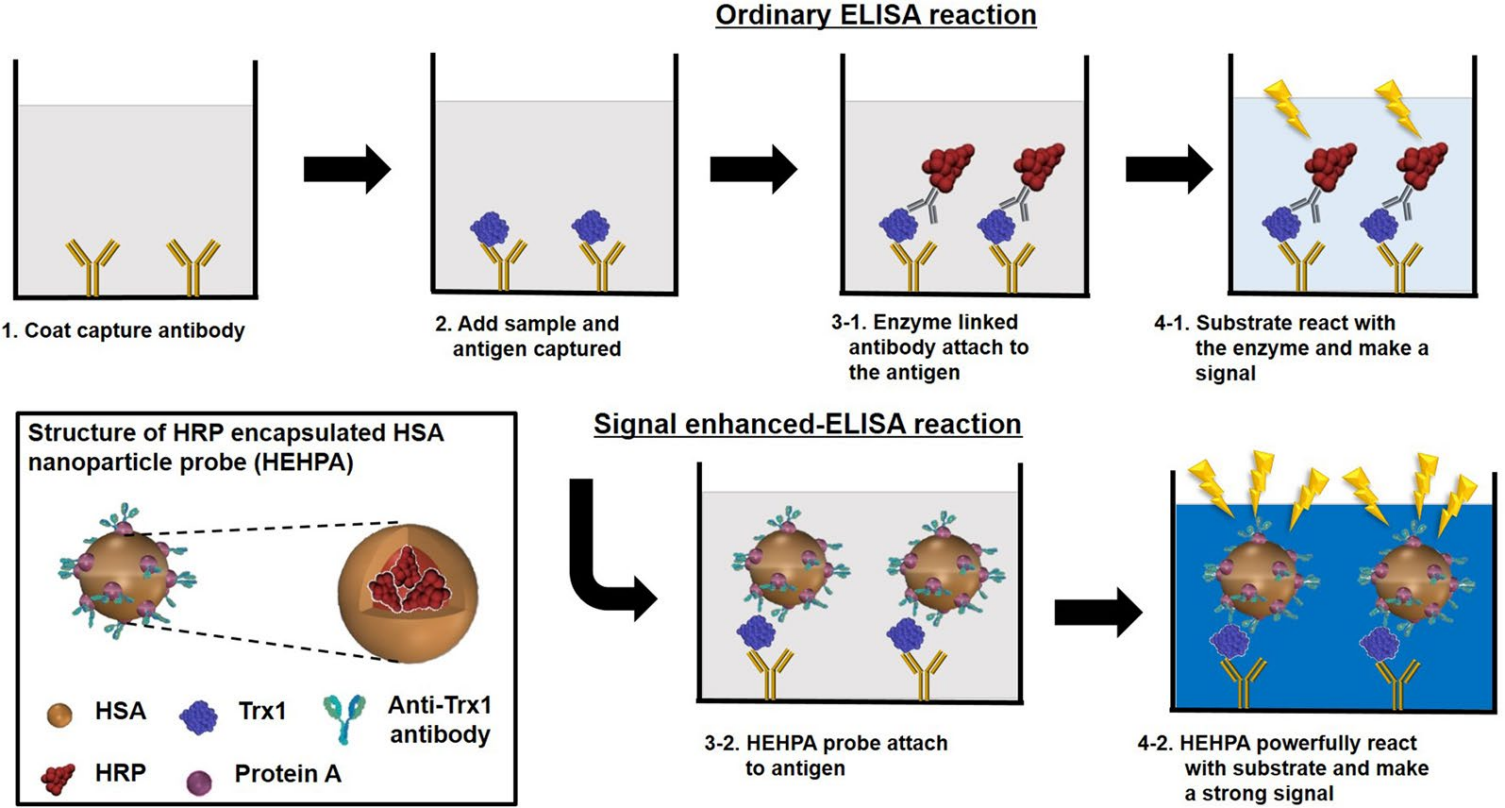
Structure of HEHPA and scheme of overall ELISA reaction steps using HEHPA for signal enhancement

A schematic diagram of the ultra-high sensitive ELISA procedure and HEHPA is depicted in Fig. 1. In the composition of HEHPA, first, HSA is used as a carrier to make protein nanoparticles capable of encapsulting large amounts of HRP. Then ProA is attached to the surface of the nanoparticle, and antibodies are attached that can detect specific antigens. The order of the overall ultra-high sensitive ELISA is not very different from that of a typical ELISA. The only difference is that when adding detection antibodies to quantify specific antigens after antigens are attached to capture antibodies on ELISA plate, the common detection antibodies has a single molecule called HRP per detection antibody, but for HEHPA there is a large amount of HRP attached to each nanoprobe antibody. This is why you can generate a much stronger signal than a common detection antibody. In addition, the ELISA signal development process using the HEHPA is exactly the same as the normal ELISA signal development process and does not require any additional signal enhancer other than TMB.

### Characteristics of HEHPA

**Fig. 2:**
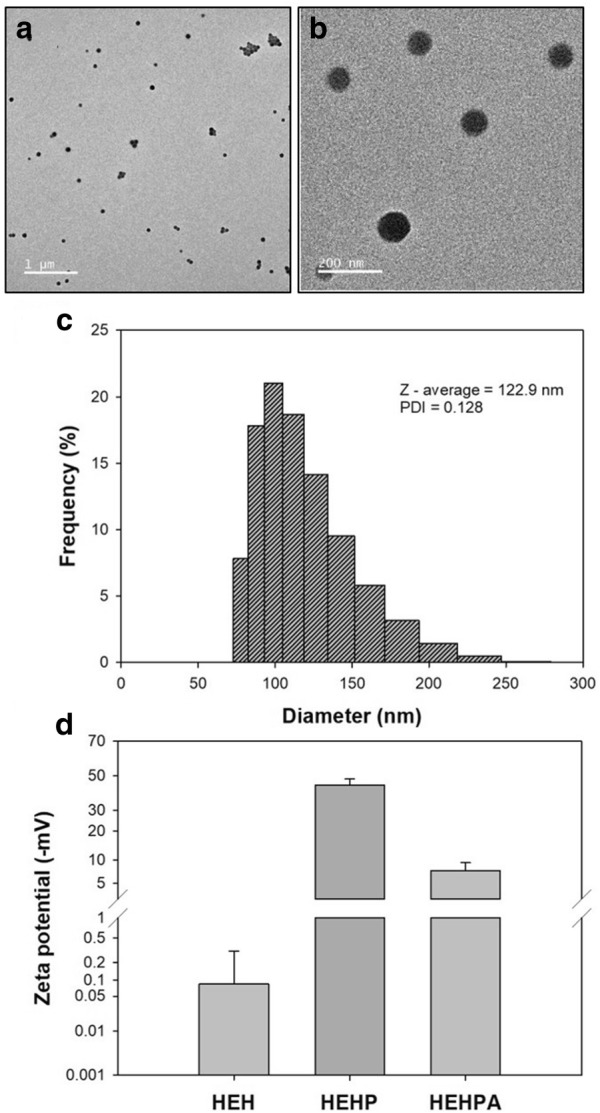
Characteristics of HEHPA. **a** and **b** TEM image of HEHPA nanoparticles (inset shows a high-resolution TEM image of randomly selected HEHPA nanoparticles), **c** DLS data of HEHPA nanoparticles and **d** zeta-potential value of each nanoparticle (n = 4) to show each component conjugated on surface of HEH

HEHPA was made by the ethanol desolvation method. It was spherical in shape, and the size was about 120 nm under synthesis condition of pH 8. The TEM images of the nanoparticles are shown in Fig. 2a, b. The uniformity of HEHPA can be seen through the TEM images, as can the even distribution of particle size (Fig. 2c). The HEHPA consists of several proteins, such as HEH, proA, and antibody. Therefore, it is necessary to verify that each protein is attached to the surface of the particles in order. Each HEHPA component protein has a different isoelectric point (pI) value (those of HSA, HRP, proA, and rabbit polyclonal antibody are 4.7 [23], 8.8 [24], 5.1, and 6.1–6.5, respectively). When the protein solution’s pH value is under the pI value, its surface charge becomes positive. Meanwhile, if the pH is greater than the pI value of the surface protein, its surface charge becomes negative. This phenomenon comes from the charge of amino acids residues, which changes depending on pH. Therefore, it is possible to estimate the surface components of HEHPA by measuring the zeta-potential value (Fig. 2d). In distilled water, pH 7, HEH’s zeta-potential value is − 0.083 mV, which is a value near zero due to the effect of HRP and HSA. After the attachment of proA on the surface of HEH, the zeta-potential value shifts to − 44.3 mV, because proA’s pI value is far from pH 7. This result shows that proA was immobilized on the surface well. HEHPA is an antibody-attached probe with a surface surrounded by antibody, and its zeta-potential value is − 7.35 mV. Because rabbit antibody has a higher pI value and is closer to pH 7 than proA, the surface charge changes. This ensures that proteins are fixed to the surface in order.

**Fig. 3:**
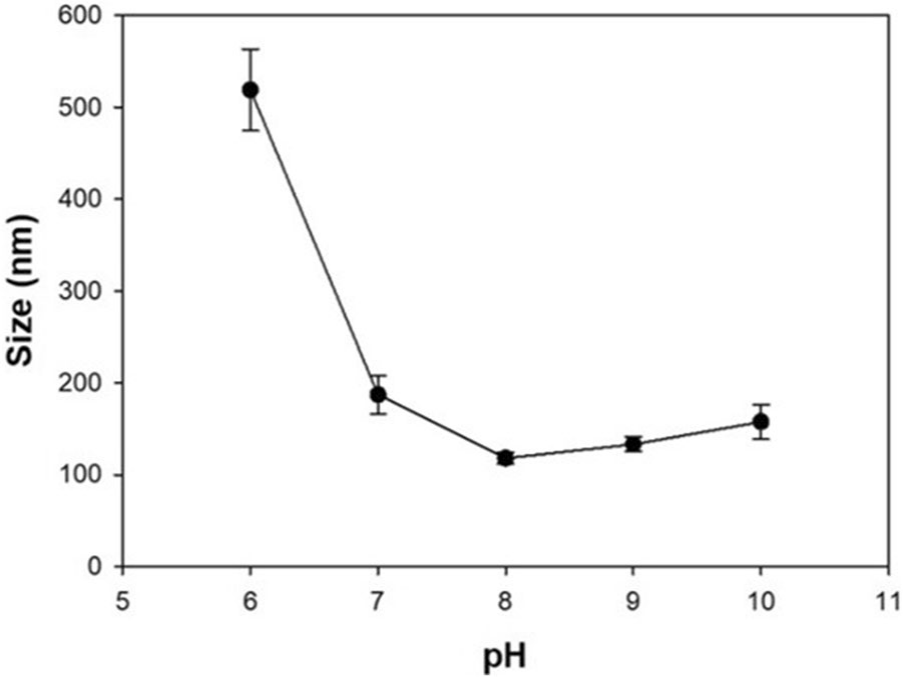
Size of HEH nanoparticle according to initial pH of HSA solution

**Fig. 4:**
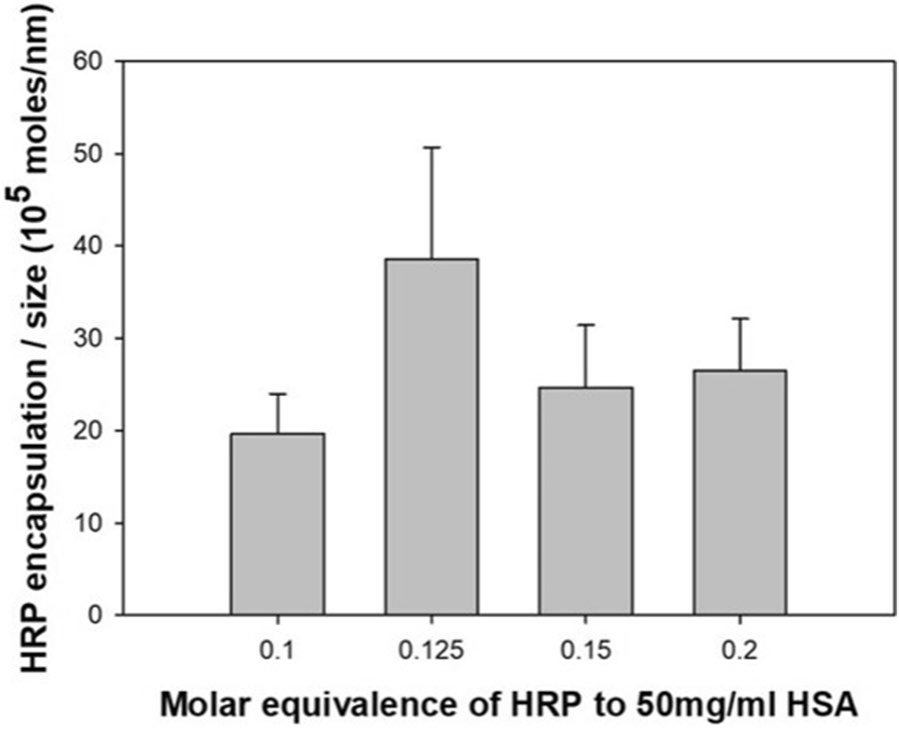
Optimization of encapsulated HRP amount per particle size related to initial molar equivalence of HRP to 50 mg/mL HSA

It is known that particles are better at producing signals when they are made smaller due to their overall surface area and reactivity. In addition, when it comes to detecting nano-sized substances such as proteins, small probes can better physically capture the target. The size of the core particle, the HEH nanoparticle, can be controlled according to the pH when synthesizing. As identified in Fig. 3, the increase in pH decreases the particle size, which can be explained with respect to the ionization of amino acid residues in HAS [25]. A higher pH value corresponds to more negatively charged residues, which induces increased repulsion among the albumin molecules and small size aggregation during the desolvation process. When the pH exceeded 8, it is seen that the particles were aggregated by the influence of the pI value of HSA. In addition, pH 8 was taken as an appropriate synthetic condition because strong alkali can dissolve or disable proteins. Signal optimization was done by looking at the size and amount of encapsulated HRP per particle. First, we fixed the initial concentration of HSA, 50 mg/mL, volume 100 μL, and pH 8, which are the optimized conditions for size. In the initial step, we changed the HRP concentration to determine the amount of HRP encapsulated in HEH. This was conducted using a four molar ratio scale (HSA:HRP = 1:0.1, 1:0.125, 1:0.15, and 1:0.2). Figure 4 shows the encapsulated HRP amount per diameter of HEH for each component ratio. As the amount of HRP increases, due to the pI value, the particle size gradually increases. However, as the size increases, the amount of encapsulated enzyme does not increase proportionately. When the molar ratio with HSA increases to over 0.125, the amount of HRP per size decreases. For this reason, over-dosing the HRP does not produce much more encapsulation in the particle but rather increases the particle size. The 1:0.125 molar ratio of HSA to HRP is the point at which there is the most HRP encapsulation per unit size.

### Colorimetric signal amplification of HEHPA

**Fig. 5:**
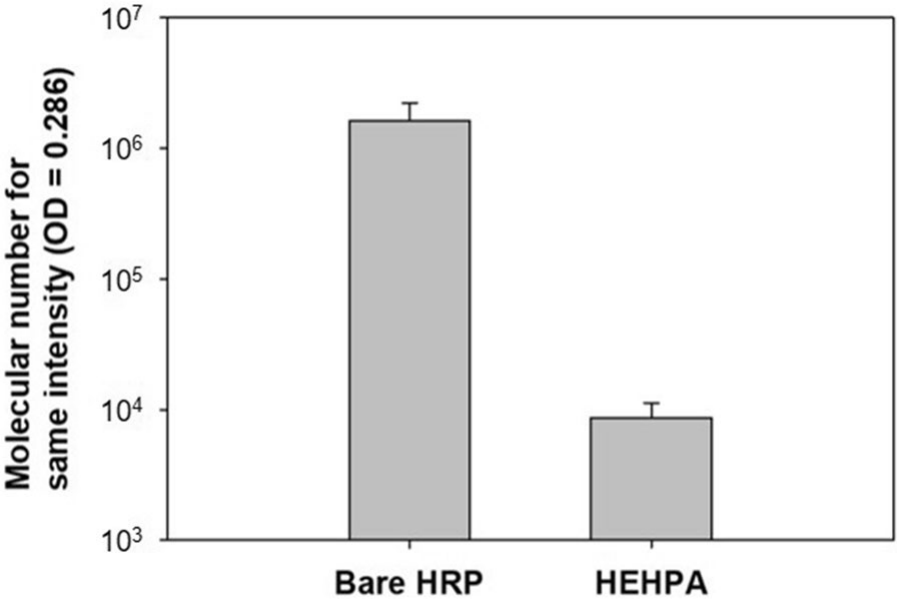
Number of nanoparticles needed to generate same signal intensity = 0.286 (OD = 450 nm) in 13 min (n = 4)

In general, HRP coupled with antibody provides excellent signal generation in conventional colorimetric assays such as direct sandwich ELISA. However, HRP bound to that antibody is not sufficient to generate a more sensitive detection signal for target proteins below pM concentration. The HEHPA developed in this study is expected to provide very sensitive detection signal in ELISA because it can provide the excess enzyme needed to attach and react to a single copy of the target protein. The size of HEHPA synthesized at a pH 8 in 1:0.125 molar ratio of HSA to HRP was about 120 nm. The particle size was the smallest and contained the most HRP in the particles and surface. Color signal amplification experiments with HEHPA show how small amounts of HEHPA can generate the same intensity color signal compared to bare HRP. The result is shown in Fig. 5. The experiment was done under the same conditions, which were 100 μL of substrate (TMB) and an enzyme reaction time of 13 min. To generate a fixed signal intensity at 0.286 in 13 min, HRP needs 1.64 × 10^6^ molecules. In contrast, HEHPA only needs 8.72 × 10^3^ molecules. This result shows that 180-fold more bare HRP is required than HEHPA to provide a signal of the same intensity. Conversely, HEHPA is able to produce signal amplification 180 times stronger than bare HRP. Therefore, the HEHPA probe makes a much more powerful reaction with the substrate compared to the bare HRP. Overall, HEHPA developed in this study was much better than bare HRP as signaling probes in ELISA kits.

### Detection of Trx1 by signal-enhanced ELISA using HEHPA

**Fig. 6:**
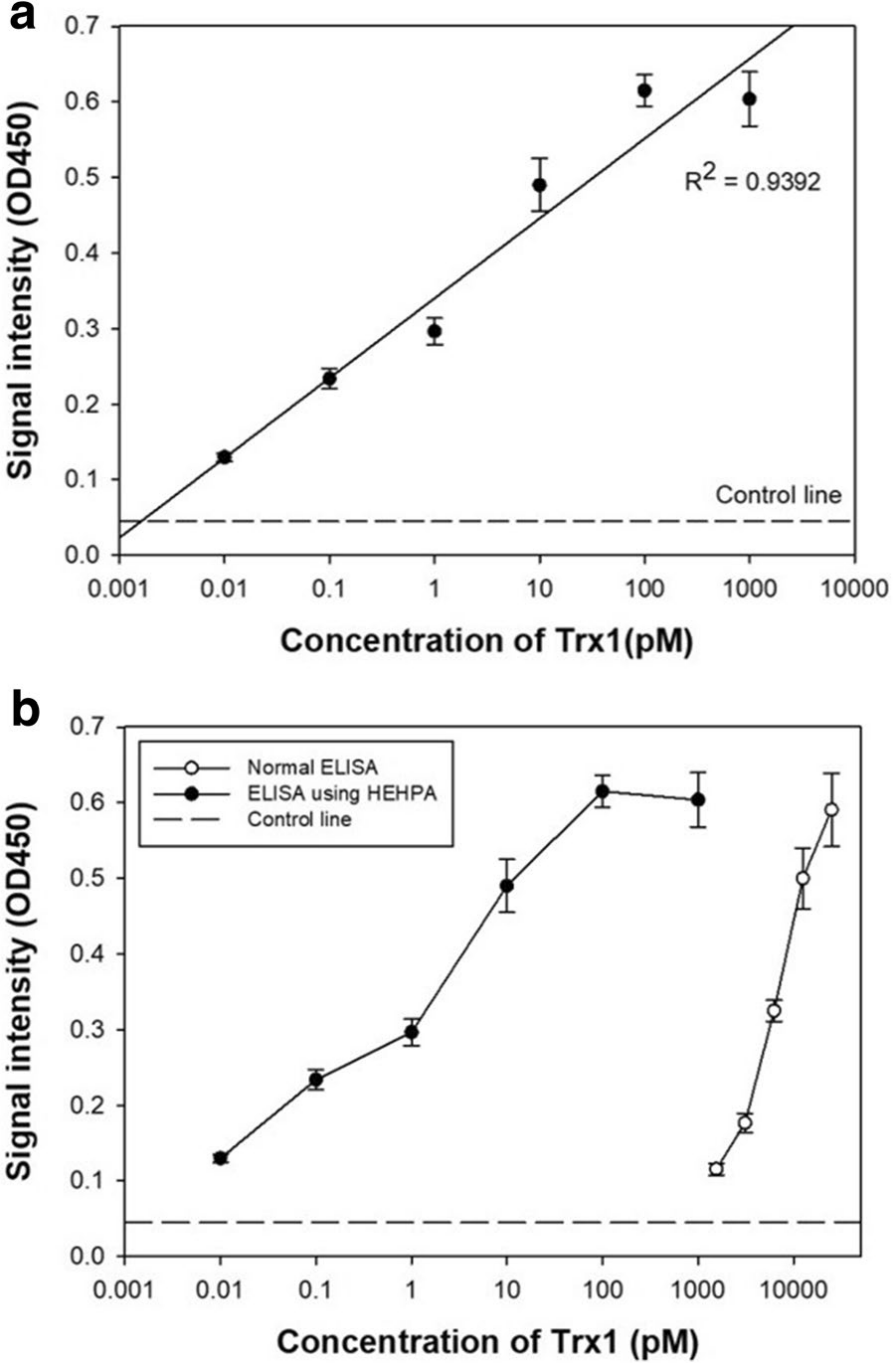
ELISA results **a** using HEHPA for detection of Trx1 to see signal amplification. **b** Comparison of ELISA using HEHPA with ordinary ELISA using bare HRP probe. All signal intensities collected at OD = 450 nm (n = 4)

The sandwich ELISA method is commonly used in the diagnosis of disease. To perform a basic ELISA, four materials are needed: capture antibody, antigen, HRP coupled detection antibody probe, and enzyme substrate. HEHPA has a role as a HRP coupled detection antibody probe because it has a large amount of HRP and antigen-tracking antibody on its surface. Using HEHPA, Trx1, which is used for the diagnosis of breast cancer, was detected by the sandwich ELISA method. Figure 6a shows the results of Trx1 detection using HEHPA, demonstrating its enhanced signal and sensitivity. This result shows that HEHPA could detect samples at ultra-low (femtomolar-scale) concentrations of Trx1. The ELISA result with the signal amplified by HEHPA exhibits a high linearity at the log scale of Trx1 concentration from 10 fM to 100 pM. For 100 pM or more, it can be seen that the signal does not increase. This is an appropriate response due to signal development saturation, which is common in ELISA reactions. From this, it could be confirmed that there is no significant difference from the signal pattern with ELISA using a general HRP. Overall, HEHPA-based ELISA could detect Trx1 in a range from 10 fM to 100 pM. In general, the measurable concentration limit of ELISA is 0.1–1 nM when using a general probe, antibodies, and HRP. In order to compare the degree of amplification of the signal according to the probe particle, an ELISA reaction was performed under the same conditions with an HRP-antibody probe and HEHPA (Fig. 6b). The HRP-based ELISA reaction can detect in a range from 1.25 to 25 nM. However, in the same conditions, HEHPA can detect 10 fM of Trx1 with a high response and a wide range of detection. The normal ELISA signal is 0.085 at 1250 pM. In contrast, the HEHPA-based ELISA signal is much higher (0.60) at 1000 pM. From this comparison, it could be confirmed that the HEHPA probe is more sensitive than the commonly used HRP probe.

## Conclusion

In this study, we synthesized a powerful signal-generating probe consisting of an encapsulated HRP-rich HSA protein nanoparticle that binds the antibody on its surface by using proA. Its size is controlled by synthesis conditions to obtain a single particle size as small as 120 nm. This nanoparticle can be applied to ELISA for detecting Trx1. Using HEHPA, it is possible to visually detect the Trx1 antigen at femtomolar concentrations ranging from 10 fM to 1 nM with high linearity at the range of 10 fM to 100 pM. HEHPA can be a useful tool to detect antigens in other serum-unenriched samples. Because these particles have proA on the outer surface, it is very easy to bind or change the antibody. Therefore, it can be applied to an HRP conjugate kit or to any other antigen detection kit. In addition, these particles capable of signal amplification with high efficiency can be applied not only to ELISA but also to various biosensors, which require highly amplified signals using peroxide enzymes, such as electrochemical sensors or immune sensors (e.g., paper-based lateral flow assay). Due to its very low detection limit, it is expected that this nano-sized enzyme nanoprobe will be a useful tool for finding relationships between unenriched biomarkers and early-stage cancer in a wide variety of diagnostic applications.

## Methods/experimental

### Materials

Albumin (from bovine serum and human serum), glutaraldehyde (25% in H_2_O), Tween 20, casein, phosphate buffer saline (PBS), Tris-hydrochloride (Tris–HCl), and TMB solution were purchased from Merck (Darmstadt, Germany). HRP and Bradford Quick Start dye (Coomassie Blue G250) were purchased from Thermo-Fisher Scientific (Waltham, USA). Ethanol (> 99%, extra pure) was purchased from Daejung Chemistry (Siheung, Korea). ProA was purchased from BioVision (Milpitas, USA). Amicon filters (3 kDa cutoff) were purchased from Merck. Transmission Electron Microscopy (TEM) grids (only carbon film, 200 mesh) were purchased from Ted-Pella (Redding, USA). Human Trx1 and IgG1-isotype anti-Trx1 antibody from mouse (ab57675) were purchased from Abcam (Cambridge, UK), and anti-Trx1 rabbit polyclonal antibody (14999-1-AP) was purchased from Proteintech (Rosemont, USA). HRP-conjugated polyclonal antibody from rabbit (MBS715155) was purchased from MyBioSource (USA).

### Synthesis of HEH

HEH is a base nanoparticle for HEHPA. These albumin nanoparticles were synthesized by using the ethanol desolvation technique [26]. First, 16 μL of 25 mg/mL HRP solution in water was added to 100 μL of pH 8, 50 mg/mL HSA solution and mixed. After mixing, 400 μL of 99% pure ethanol was added dropwise to the solution with a flow rate of 1 mL/min while being stirred at 900 rpm, 25 °C. After 10 min, 11.7 μL of 4% glutaraldehyde solution was added for crosslinking the albumin and HRP [27]. The mixture was stirred for 12 h. After the reaction finished, a washing step was done by centrifugation (9000 rpm, 15 min), and the supernatant was removed. The protein pellet was dispersed by 500 μL of the washing solution three times, which was composed of 0.01 M PBS with 0.01% Tween 20. After the final centrifugation, the protein pellet was dispersed by 100 μL of 1% bovine serum albumin (BSA) in Tris-buffer to deactivate glutaraldehyde. The HEH was stored at 4 °C in a dark room.

### Immobilization of antibody on surface of HEH

To form the probe, proA was used for conjugating the antibody and HEH. The conjugation of proA on the surface of HEH was conducted by the following method. First, HEH’s buffer was changed to distilled water. Then, 28.8 μL of proA solution was added to 200 μL of HEH solution, and 11.7 μL of 4% glutaraldehyde was added for crosslinking between proA and the particles (forming the so-called HEHP). After the mixture was incubated overnight with mixing, a washing step was performed twice using a washing solution, and the mixture was stored in 1% BSA Tris-buffer solution (pH 8.0). To the HEHP mixture, 4.2 μL of rabbit host anti-Trx1 antibody was added, and it was incubated at 4 °C for 3 h. Finally, the product was washed with washing buffer and stored in PBS at 4 °C until use; the resulting nanoparticles were the HEHPA.

### Characterization of nanoparticles

The size and uniformity of HEHPA were measured using dynamic light scattering (DLS [SZ-100, Horiba, Japan]). The sample for DLS was diluted tenfold in deionized water. In addition, using the same instrument, the zeta-potential was measured for the surface characterization of HEH, HEHP, and HEHPA. In that case, all of the sample solvent was substituted with distilled water and diluted ten-fold using the distilled water (pH 7). The encapsulation efficiency of HEHPA was evaluated by quantifying the non-encapsulated HRP and manually counting the complete HEHPA. The former was conducted via the TMB reaction in supernatants, which were ultra-filtrated with an Amicon filter, and the latter was conducted via TEM analysis (JEM-F200, JEOL, Japan) [28].

### Detection of Trx1 by direct sandwich ELISA

The ELISA test was conducted by applying the HEHPA as follows: the capture antibody (anti-Trx1 antibody from mouse) was coated on the 96-well plate (5 μg/mL, 100 μL) for 4 h at 24 °C and washed four times using a washing buffer (0.1% Tween 20 in PBS). The plate surface was blocked by blocking buffer (3% w/v BSA in PBS) to avoid non-specific reactions. After 2 h at 24 °C, it was washed four times using a washing buffer. Then, samples of known concentration were added, and the plate was incubated for 1 h in a slowly shaking incubator. After that, it was washed four times. The pre-made HEHPA detection probe or antibody-HRP probe was added and incubated for 30 min. After washing five times, the TMB solution was added as a substrate of the HRP. Then, 13 min later, the reaction was stopped by using 1 M HCl, and the signal intensity was read using a microplate reader.

## Data Availability

The datasets used and/or analyzed during the current study are available from the corresponding author on reasonable request.

## Abbreviations

ELISA: enzyme linked immunosorbent assay
HSA: human serum albumin
HRP: horseradish peroxide
TMB: 3,3′,5,5′-tetramethylbenzidine
Trx-1: thioredoxin-1
ProA: proteinA
HEH: HRP encapsulated HSA nanoparticles
TEM: transmission electron microscopy
